# Significance of biomarkers in stewardship program in pediatric patients infected with *Aspergillus* species

**DOI:** 10.1186/s13052-022-01306-6

**Published:** 2022-06-25

**Authors:** Parisa Badiee, Ali Amanati, Fatemeh Ghasemi, Hadis Jafarian

**Affiliations:** Clinical Microbiology Research Center, Shiraz University of Medical Sciences, Shiraz, Iran

**Keywords:** Invasive aspergillosis, Pediatrics, Procalcitonin, sTREM-1, C-reactive protein

## Abstract

**Background:**

The potential use of biomarkers in the diagnosis of fungal infections is a challenge. The aim of this study was to evaluate the role of a biomarker-guided antifungal stewardship program for hospitalized pediatrics suffering from invasive aspergillosis (IA).

**Methods:**

Pediatric patients with suspected probable or proven IA were enrolled in this study. Demographic data were collected from their records. Clinical samples were examined by wet mount KOH smear, culture, galactomannan Ag test, and real-time PCR. Patients’ sera were evaluated for procalcitonin (PCT) and soluble-triggering receptor expressed on myeloid cells -1 (sTREM-1) levels by ELISA Kits.

**Results:**

A total of 73 children were entered in this study with a mean age of 5 years and the male to female ratio 39/34. The most predisposing factors were hematologic disorders (71.2%). The area under the curves (95% confidence interval) for each biomarker were 0.9 (0.85% to 97%) for lactate de hydrogenase (LDH), 0.9 (0.85% to 0.94%) for C-reactive protein, 0.8 (0.75% to 0.84%) for PCT, 0.8 (0.73% to 0.85%) for erythrocyte sedimentation rate, 0.7 (0.6% to 0.8%) for sTREM-1, and 0.5 (0.45% to 0.58%) for white blood cell count. During the study period, 27.4% patients died. The LDH and sTREM-1 levels were significant increase in died patient (*p* < 0.05).

**Conclusions:**

According to our data, evaluation of biomarkers along with radiologic and clinical signs and symptoms of pediatric patients can lead to proper antifungal therapy and decreased side effects, antifungal resistance, and cost. The combined measurements could be better than a single marker in the prediction of IA.

## Background

Parallel with an increased number of immunocompromised patients, the incidence and burden of fungal infections have increased in recent years. The rate of proven and probable invasive fungal infections in immunocompromised pediatric patients was reported at 42% (26/62) [1] with a mortality rate of 50% [2]. The risk factors for these infections are hematologic malignancies (acute myelogenous leukemia), bone marrow and solid organ transplantation, a regimen of high-dose chemotherapy and immunosuppression, corticosteroid therapy, persistent neutropenia, cytomegalovirus, hyperglycemia, and AIDS [1–3]. Invasive aspergillosis (IA) is the most prevalent mold fungal infection in pediatrics [3] with signs and symptoms similar to viral and bacterial infections. Therefore, distinguishing between these infections is critical in the management of the respective patients. Early diagnosis of systemic aspergillosis with early suitable antifungal therapy plays a fundamental role in the outcome of infected patients. Reliable diagnostic tests are limited and the results should be interpreted in combination with clinical and radiological evidence. The gold standard diagnostic test is the isolation of etiologic agents from the sterile clinical samples or presents the invasive form of fungi in the pathology smears. The sensitivity of fungal culture is low and needs about two weeks for the isolation of fungi. In contrast, molecular diagnostic tests (e.i. PCR) and evaluation of *Aspergillus* antigen test (i.e. galactomannan, GM) are rapid and sensitive diagnostic methods but not available in routine labs in many regions.

Biomarkers present valuable and accurate information about individual medical conditions and can be used as the basis for the diagnosis methods. Procalcitonin (PCT) is produced by the liver, C cells of the thyroid gland, K cells, neuroendocrine lung cells, and monocytes (5). A Soluble-triggering receptor expressed on myeloid cells (sTREM) plays an important role in the regulation of bacterial and fungal inflammatory responses [4]. In febrile patients with cancer, a significant association between fungemia/ bacteremia and sTREM-1 levels was reported [5]. Lactate dehydrogenase (LDH) levels in the blood and body fluid samples rise in the cell damage (the brain, muscles, kidney, and other humans cells). C reactive protein (CRP) value, erythrocyte sedimentation rate (ESR), and white blood cell (WBC) count are the indexes in the diagnosis of inflammation and infections.

Early identification of IA can lead to suitable antifungal treatment with a higher chance of survival for the patients. Empirical or prophylactic antifungal therapy was recommended in suspected patients. Antifungal stewardship programs need worldwide attention to optimize the antifungal used to promote patient care. Evaluation of the biomarkers can help prompt the diagnosis of IA, improve outcomes for the patients, and decrease the adverse events, costs, and antifungal resistance associated with the use of unnecessary antifungal therapy. There is limited data available on the role of biomarkers in the diagnosis of IA in pediatric patients; therefore, the current study aims to evaluate the role of biomarkers in the diagnosis and antifungal stewardship programs for hospitalized pediatrics suffering from IA.

## Methods

### Study population

In this cross-sectional study, from June 2017 to June 2020, immunocompromised pediatric patients with suspected probable or proven IA admitted to Shiraz University Hospitals, southern Iran, were enrolled in this study. Sampling was a part of the diagnosis process. Patients without a definitive diagnosis were excluded from the study. Eighteen healthy individuals were entered in this study as control and their sera were evaluated with similar methods for biomarker assays. Patients’ data including age, sex, background disease, clinical signs and symptoms, fever, blood biomarkers (WBC count, CRP, ESR, and LDH values), site of infection, histopathological evidence, antifungal therapy received for the previous treatment or prophylaxis, and outcomes were collected from their corresponding records. The diagnosis of infection was established based on clinical signs and symptoms, radiological, and mycological lab findings of IA, according to the European Organization for Research and Treatment of Cancer and the Mycoses Study Group (EORTC/MSG) [6].

### Mycological study

Clinical samples including sputum, bronchoalveolar fluid, wound, tissue, and pleural effusion were examined by wet mount KOH smear and cultured in Sabouraud dextrose agar medium (Merck, Germany). The identification of the *Aspergillus* (*A*) spp. was achieved by macroscopic and microscopic evaluation of the isolates. Circulating *Aspergillus* DNA in the blood and other clinical samples were extracted and detected by real-time PCR [7]. Galactomannan Ag test (PlateliaTM Ag assay; Bio-Rad, Germany) was performed in blood samples and/or bronchoalveolar washing fluids of pediatrics, according to the manufacturer’. Lab procedures were done in biological safety cabinet class 1 to avoid contamination.

### ELISA identification

The PCT and sTREM-1 levels of patients were evaluated by ELISA Kits (Shanghai crystal Day Biotech co. LTD, China), according to the manufacturer’s instructions.

## Statistical analysis

Data analysis by using SPSS version 16. The data were not normal, according to Kolmogorov–Smirnov and Shapiro–Wilk tests. Mann–Whitney test was used to compare the variables and evaluate differences between the groups. The receiver operating characteristics (ROC) curves, sensitivity, and specificity of each biomarker were analyzed using the statistical package, GraphPad Prism. Statistical significance was set at the p < 0.05.

## Results

A total of 73 children with proven and probable IA were entered in this study. The mean age of patients was 5 years (range:10 months to > 18 years) and the male to female ratio was 39/34 (53.4%/46.6%). Hematologic disorders were the most predisposing factors 52 (71.2%) and the lung was the most site of infections (38/73, 52%). Forty-three patients (58.9%) had received antifungal agents as prophylaxis or the treatment of previous fungal infections (Table 1).

**Table 1:** Clinical characteristics of the pediatric patients with invasive aspergillosis in Shiraz, southern Iran

Demographic features	
Male/female (%)	39/34 (53.4%/46.6%)
Age mean (range)	5 years (10 month > 18 years)
Underlying Disease	
Hematologic disorders	52 (71.2%)
Heart surgery	8 (11%)
Liver Surgery	6 (8.2%)
Immunodeficiency disease	5 (6.8%)
AIDS	1 (1.4%)
Diabetes	1(1.4%)
Site of infection	
Lung	38 (52%)
Liver	24 (32.9%)
Heart	7 (9.6%)
Sinuses	5 (6.8%)
Kidney	2 (2.7%)
Fever of unknown origin	9 (12.3%)
Antifungal therapy	
Not used	30 (41.1%)
Amphotericin B	9 (12.3%)
Itraconazole	4 (5.5%)
Voriconazole	3 (4.1%)
Fluconazole	8 (11%)
Combination therapy^a^	19 (26%)
Outcome	
Alive	53 (72.6%)
Death	20 (27.4%)

^a^Combination of amphotericin B and voriconazole with caspofungin or amphotericin B, and Fluconazole

*Aspergillus* species were isolated from clinical samples of 42 patients. Eleven *Aspergillus fumigatus* were isolated from the bronchoalveolar lavage fluids, sputum, and central nervous system, 28 *Aspergillus flavus* from sputum, sinuses tissue, and bronchoalveolar lavage fluids, and three *Aspergillus niger* from sputum and bronchoalveolar lavage fluids. 255 sera from 18 healthy volunteers (*n* = 36) and 73 patients suffering from probable and proven IA (*n* = 219) were entered into the study. Galactomannan and PCR were positive in the study population.

**Fig. 1:**
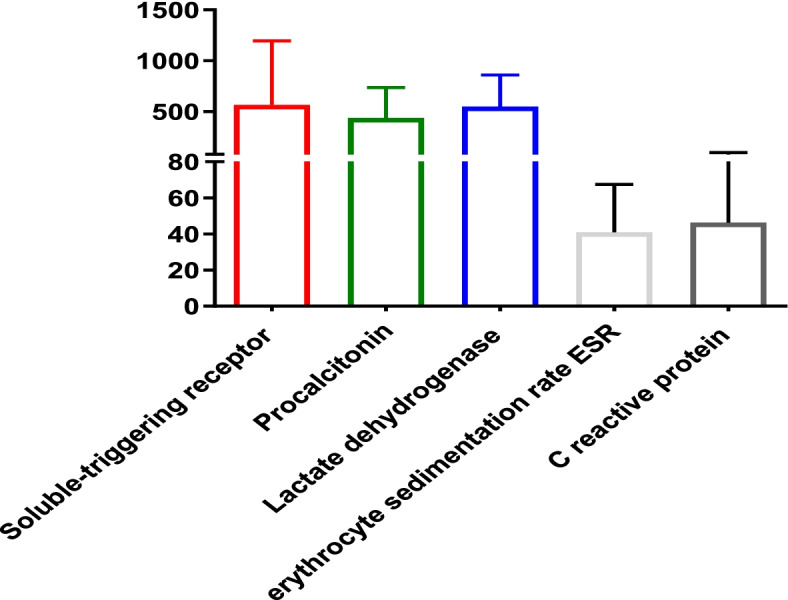
Median of biomarkers in patients suffering from invasive aspergillosis

**Fig. 2:**
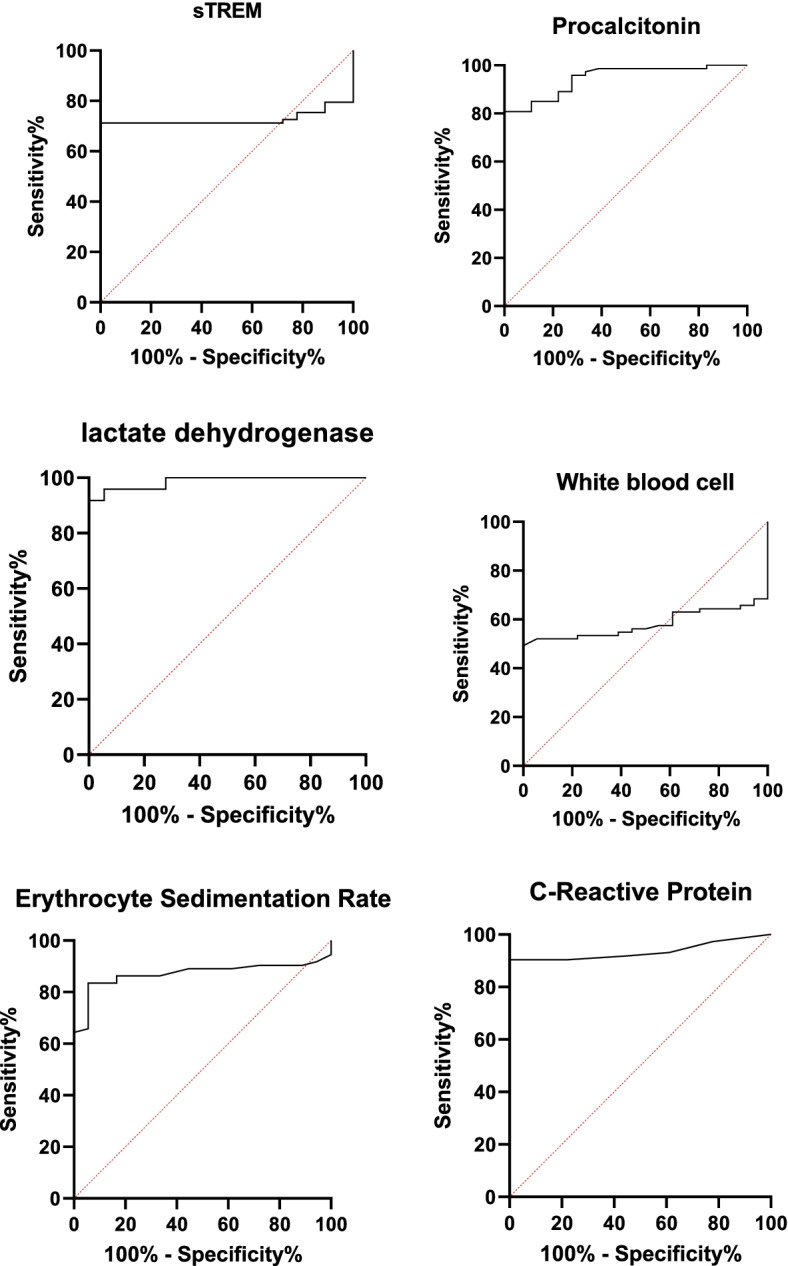
Receiver operating characteristics curves for evaluating the diagnostic role of biomarkers in patients with invasive aspergillosis

**Fig. 3:**
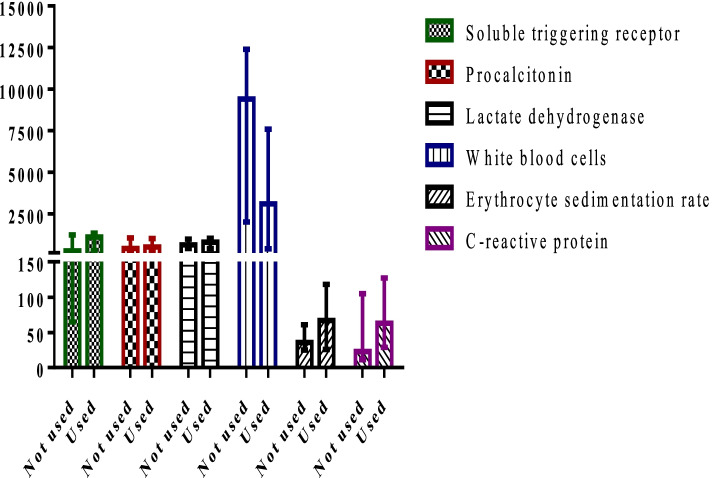
A comparison chart of evaluated biomarkers in patients who have received antifungal agents and those who have not

**Fig. 4:**
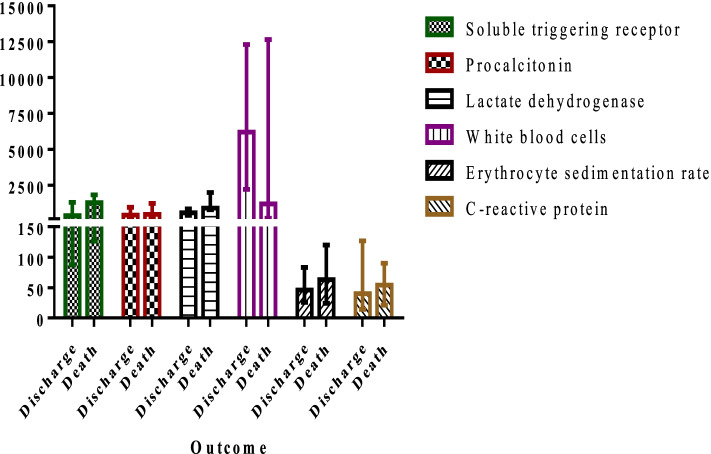
A comparison chart of evaluated biomarkers in living and dead patients

The median (interquartile range 1 and 3, IQR) values of sTREM-1, PCT, CRP, ESR, LDH, and WBC count in patients were 291.2 (124.8, 1286.8) pg/ml, 362.4 (185.6, 768.8) pg/ml, 29 (5–105) mg/L, 35 (18, 70) mm/h, 562 (235, 854) U/L, and 6590 (1500, 9800) cells/ml, respectively (Fig. 1). Table 2 presents the area under the curve (AUC), cut-off values, sensitivity, specificity, positive predictive values and negative predictive values of biomarkers in infected pediatric patients. The cut-off values (sensitivity, specificity) for sTREM-1, PCT, LDH, CRP, ESR, and WBC count in patients were observed > 190 pg/mL (71.2%, 100%), > 260 pg/mL (80.8%, 100%), > 252 U/l (91.8%, 100%), > 7 mg/l (90.4%, 100%), > 23 mm/h (83.6%, 94.4%), and ≤ 4930 cells/l (49.3%, 100%), respectively. The AUC (95% confidence interval) for each biomarker were 0.9 (0.85% to 0.97%) for LDH, 0.8 (0.75% to 0.84%) for PCT, 0.9 (0.85% to 0.94%) for CRP, 0.8 (0.73% to 0.85%) for ESR, 0.7 (0.6% to 0.8%) for sTREM-1, and 0.5 (0.45% to 0.58%) for WBC count (Fig. 2). There was a significant relationship between sTREM-1 and PCT (p = 0.001), and CRP values (*p* = 0.02) in diagnosis of IA and all of these biomarkers elevated during infection. In this study 43 patients received antifungal agent as prophylaxis or treatment of previous fungal infections (Fig. 3). The ESR, CRP and sTREM-1 values were increased and WBC count decreased in patients using antifungal agents. During the study period, 20 patients died (27.4%). The LDH and sTREM-1 level were significant increase in died patient (*p* < 0.05) (Fig. 4).

**Table 2:** The area under the curve (AUC), cut-off values, sensitivity, and specificity for biomarkers invasive aspergillosis cases

	sTREM-1	Procalcitonin	Lactate dehydrogenase	C-reactive protein	White blood cells	Erythrocyte sedimentation rate
AUC	0.7	0.8	0.9	0.9	0.5	0.8
95% CI^a^	0.6% to 0.8%	0.75% to 0.84%	0.85% to 97%	0.85% to 0.94%	0.45% to 0.58%	0.73% to 0.85%
*P*-value	0.003	< 0.0001	< 0.0001	< 0.0001	0.29	< 0.0001
Cut- off Value	> 190	> 260	> 252	> 7	≤ 4930	> 23
Sensitivity	71.2%	80.8%	91.8%	90.4%	49.3%	83.6%
95% CI	59% to 81%	70% to 88.2%	83.2% to 96.2%	81% to 96%	37% to 61%	73% to 91%
Specificity	100%	100%	100%	100%	100%	94.4%
95% CI	81.5% to100%	81.5% to100%	81.5% to100%	81.5% to100%	81.5% to100%	72.7% to 99.9%
PPV^a^	100.0	100.0	100.0	100.0	100.0	98.4
NPV^a^	46.2	56.2	75	72	32.7	58.6

^a^*CI* confidence interval, *PPV* Positive Predictive Value, *NPV* Negative Predictive Value

## Discussion

Antifungal stewardship programs can save a patient’s quality of life by avoiding unnecessary empirical or prophylactic antifungal therapy. Early diagnosis and best management of infection are parts of the stewardship program. Diagnosis of fungal infection is not easy and respective clinical signs and symptoms are similar to those of other bacterial and viral infections. The antifungal resistant species are reported in the literature [8, 9]. Appropriate treatment with antifungal drugs can lead to decreased side effects, fungal resistance, and cost. In critically ill patients and where only routine lab exists and specific molecular and serological methods are not available, the use of biomarkers values can help diagnose IA and proper empirical antifungal therapy. Some of the biomarkers are easily accessible in clinical settings for regular monitoring. Evaluation of the biomarkers including PCT and sTREM-1, WBC counts, ESR, LDH, and CRP values can promote the diagnostic accuracy of IA.

The TREM-1 is expressed on myeloid cell-1 response to bacterial and fungal infections, with its soluble form (sTREM-1) released during human infected tissue [10]. According to the literature, in febrile infants and neonates, the level of sTREM-1 in plasma could be considered a valuable marker for early diagnosis of sepsis [11]. Unfortunately, only limited data are available on the role of sTREM-1 and its cut-off value in the diagnosis of IA in pediatrics. sTREM-1 cut-off value of 300 pg/mL with a sensitivity of 0.78 and specificity of 0.97 was reported for septic shock and death in neonates [11]. Aksaray and co-workers reported the cut-off value ≥ 133 pg/mL was significant as a diagnostic biomarker of sepsis with a sensitivity of 71.1% and specificity of 73.3% [12]. In the present study, 69.2% of infected pediatrics had sTREM-1 levels higher than 133 pg/mL. Procalcitonin is a precursor of calcitonin and a part of the innate immune system. There is an association between PCT level and the severity of the disease. Children with signs and symptoms of bacterial infections or pneumonia had elevated PCT levels [13]. The PCT level ≥ 0.5 µg/l (500 pg/l) was reported significant in children with severe bacterial pneumonia [13] and the level in patients with invasive fungal infection was not increased in Stoma et al. [14]. C reactive protein is an acute-phase protein that increased in any inflammatory condition and in patients with invasive fungal infections [14]. Marková et al. in a cohort study of the hematological patients reported the phenomenon of “low PCT and high CRP” in case of fungal infections [15]. Martini and coworkers in a study on surgical patients at risk of candidemia reported: “low PCT value in a critically ill septic patient is more likely to be related to candidemia than to bacteremia” [16]. In the present study, CRP level increased in 89.7% of cases while PCT increased in 51.3% consistent with the study by Gunasekaran and coworkers that reported “PCT was elevated in nearly half of documented viral and fungal infections” in pediatric oncology [17]. According to Marková et al., a combination of PCT < 0.5 μg/L and CRP 100–300 mg/L offers the best specificity and sensitivity for the diagnosis of fungal infections in immunocompromised patients [15]. There are limited data about the ESR and LDH values and WBC count in the diagnosis of IA in pediatrics. Neutropenia (< 100/μL) and prolonged neutropenia period (> 12–15 d) can create significant risk factors for developing IA in immunocompromised patients [18]. In the current study, ESR, CRP values, and WBC count were within the normal range in a few patients.

The early diagnosis and rapid initiation of effective antifungal therapy are critical for the proper management of IA, as recommended by the ESCMID-ECMM-ERS-ECMM-ERS guideline and Infectious Diseases Society of America [17, 18]. In the diagnosis of IA, microscopy, and culture of clinical samples should be attempted in immunocompromised pediatrics at risk for IA. In case of difficulty in obtaining the appropriate specimens, the use of appropriate biomarkers and interpretation of them can help early and accurate diagnosis and improve the outcomes and at the same time limit the unnecessary use of antifungal agents. According to the present data, increasing values of sTREM-1, PCT, and CRP can lead to a diagnosis of IA in pediatric patients without other etiologic agents of infection, and increasing in LDH and sTREM-1 levels present the severity of the infection and death.

The limitation of this study was entering patients with proven and probable invasive aspergillosis. Therefore, the number of patients was limited. We hope that larger and various populations are investigated in future studies. We hope that larger and various populations are investigated in future studies. Also, it is suggested that researchers use patients with no history of antifungal use in further studies.

## Conclusion

According to our data, the evaluation of biomarkers can serve as a valuable tool for the early detection of IA in pediatric patients. Evaluation of the above-mentioned factors along with radiologic and clinical signs and symptoms of pediatric patients can lead to proper antifungal therapy and decreased side effects, fungal resistance, and cost. We recommend that the combined measurement of biomarkers would be better than a single marker in the prediction of IA.

## Data Availability

All data generated or analyzed during this study are included in this published article.

## Abbreviations

IA: Invasive aspergillosis
PCT: Procalcitonin
sTREM-1: Soluble-triggering receptor expressed on myeloid cells-1
LDH: Lactate Dehydrogenase
CRP: C Reactive Protein
ESR: Erythrocyte Sedimentation Rate
WBC: White Blood Cell
EORTC/MSG: European Organization for Research and Treatment of Cancer and the Mycoses Study Group
